# Nitrates and bone turnover (NABT) - trial to select the best nitrate preparation: study protocol for a randomized controlled trial

**DOI:** 10.1186/1745-6215-14-284

**Published:** 2013-09-08

**Authors:** Roxana C Bucur, Lauren S Reid, Celeste J Hamilton, Steven R Cummings, Sophie A Jamal

**Affiliations:** 1 Women’s College Research Institute, Women’s College Hospital, Toronto, Ontario, Canada; 2 Women’s College Research Institute, Women’s College Hospital, The University of Toronto, Toronto, Ontario, Canada; 3 San Francisco Coordinating Centre, San Francisco, CA, USA; 4 Women’s College Research Institute and Department of Medicine, Women’s College Hospital, The University of Toronto, Toronto, Ontario, Canada

**Keywords:** Organic nitrates, Bone turnover markers, Postmenopausal osteoporosis

## Abstract

**Background:**

Organic nitrates uncouple bone turnover, improve bone mineral density, and improve trabecular and cortical components of bone. These changes in turnover, strength and geometry may translate into an important reduction in fractures. However, before proceeding with a large fracture trial, there is a need to identify the nitrate formulation that has both the greatest efficacy (with regards to bone turnover markers) and gives the fewest headaches. Ascertaining which nitrate formulation this may be is the purpose of the current study.

**Methods and design:**

This will be an open-label randomized, controlled trial conducted at Women’s College Hospital comparing five formulations of nitrates for their effects on bone turnover markers and headache. We will recruit postmenopausal women age 50 years or older with no contraindications to nitroglycerin. Our trial will consist of a run-in phase and a treatment phase. We will enroll 420 women in the run-in phase, each to receive all of the 5 potential treatments in random order for 2 days, each with a 2-day washout period between treatments. Those who tolerate all formulations will enter the 12-week treatment phase and be randomly assigned to one of five groups: 0.3 mg sublingual nitroglycerin tablet, 0.6 mg of the sublingual tablet, a 20 mg tablet of isosorbide mononitrate, a 160 mg nitroglycerin transdermal patch (used for 8 h), and 15 mg of nitroglycerin ointment as used in a previous trial by our group. We will continue enrolment until we have randomized 210 women or 35 women per group. Concentrations of bone formation (bone-specific alkaline phosphatase and procollagen type I N-terminal propeptide) and bone resorption (C-telopeptides of collagen crosslinks and N-terminal crosslinks of collagen) agents will be measured in samples taken at study entry (the start of the run in phase) and 12 weeks. Subjects will record the frequency and severity of headaches daily during the run-in phase and then monthly after that. We will use the ‘multiple comparisons with the best’ approach for data analyses, as this strategy allows practical considerations of ease of use and tolerability to guide selection of the preparation for future studies.

**Discussion:**

Data from this protocol will be used to develop a randomized, controlled trial of nitrates to prevent osteoporotic fractures.

**Trial registration:**

ClinicalTrials.gov Identifier: NCT01387672. Controlled-Trials.com: ISRCTN08860742.

## Background

Organic nitrates, medications used to treat angina, have the potential to reduce the morbidity, mortality and costs associated with osteoporotic fractures. The comparative effects of available nitrate formulations have not been studied. Their effects on bone formation and resorption have not been compared. Their effects on headaches, the most common adverse effect that limits their use, have not been evaluated. Therefore, we propose to compare several widely available nitrate formulations for the efficacy on markers of bone formation and resorption and for the number and severity of headaches they cause.

We have found that application of 15 mg of nitroglycerin ointment at night has unique and substantial effects on bone. Our work has demonstrated that nitroglycerin uncouples bone turnover: it increases markers of bone formation and decreases bone resorption [1]. This uncoupling leads to substantial increases in bone density, improvements in bone geometry, and increases in indices of bone strength by peripheral quantitated computed tomography (pQCT). These effects are stronger than have been observed for current treatments including bisphosphonates and parathyroid hormone.

Nitrates are an attractive therapy for osteoporosis because they are inexpensive and universally available. Further, in contrast to currently available treatments (which affect trabecular bone and primarily result in a decrease in vertebral fractures), our preliminary data suggest that nitrates also increase cortical bone density and strength. As such, nitrates, more than the available therapies, have the potential to decrease the fracture rates that occur in cortical bones (such as the hip, leg, forearm, and upper arm). These fractures are extremely common and are associated with disability and death.

A randomized trial with fracture endpoints is essential to establish the efficacy of nitrates for clinical use. Designing a fracture-prevention trial requires choosing a preparation and dose of nitrate that maximizes its beneficial effects on bone while minimizing adverse effects. Several nitrate formulations are already widely available and several are generic. As such, it is highly unlikely that a pharmaceutical company will invest in the development and clinical testing of available formulations. Thus, we must rely on Canadian Institutes of Health Research (CIHR) and other agencies' funding to select best preparation and test its efficacy and safety.

Before proceeding with a fracture trial, it is critical to identify the nitrate formulation that gives the greatest effects on bone turnover, with the fewest headaches, and is easy to use. After systematically considering the available formulations and doses, we propose to test five nitrates: 15 mg of nitroglycerin (NTG) ointment nightly (whose efficacy and safety we have established), 0.3 mg and 0.6 mg of sublingual NTG, 20 mg of isosorbide mononitrate (ISMO), and 160 mg of glycerol trinitrate (Nitro Dur), a patch worn overnight for 8 h.

In a randomized trial with 210 postmenopausal women, we will ask 2 principal research questions: (1) What are the effects of 5 formulations and doses of nitroglycerin on 2 markers of bone formation (serum bone specific alkaline phosphatase (BALP) and procollagen type I N-terminal propeptide (P1NP)) and 2 markers of bone resorption (serum C-telopeptide (CTX) and urine N-telopeptide (NTX)) over 12 weeks? And (2) How do the five formulations and doses of nitroglycerin compare with regard to frequency and severity of headaches?

We hypothesize that that there are one or two formulations that are less messy, more convenient to use, result in a similar or lower rate of headaches, and increase markers of bone formation and decrease markers of resorption at least as much as the ointment. We expect to arrive at a consensus about the best preparation to use in larger clinical trials designed to test the efficacy of nitrates for fracture prevention.

Osteoporosis (OP) is characterized by a reduction in bone mass and disruption of skeletal microarchitecture leading to an increased susceptibility to fracture with minimal trauma. OP fractures are costly to treat, cause pain, disability, and occasionally, premature death [2]. In Canada, one in four women and one in eight men have OP and in 1993 the total expenditure for fractures was in excess of CA$1.3 billion [3,4]. The average length of stay in an acute care hospital after a hip fracture is 3 weeks; one in four patients remain in long-term care institutions for at least 1 year; one in three returns home, but must depend on other people or devices for mobility. Further, after a hip fracture there is up to a 20% increased risk of mortality. As older men and women are the fastest growing group in the world and the frequency of OP fractures increases exponentially with age, the number of men and women with OP fractures is expected to increase dramatically over the next 50 years in Canada and worldwide [5]. For example, if we assume, based on current demographic trends, that the age-adjusted frequency rates of hip fracture stabilized in Europe and North America but rose by 3% per year in the rest of the world, the frequency of hip fractures worldwide could exceed 21 million in 2050 [6]. The most effective way to moderate increases in healthcare costs, sickness, and premature death associated with OP fractures is to prevent OP.

Current medications include the antiresorptives: estrogen replacement therapy (ERT), bisphosphonates (alendronate, risedronate, zoledronic acid, and etidronate), selective estrogen receptor modulators (raloxifene) and denosumab, which is a fully human monoclonal antibody to the receptor activator of nuclear factor-κB ligand (RANKL). These medications decrease bone resorption, which slows or prevents bone loss. All of these agents also decrease bone formation. Consequently, they have little effect on the thickness and strength of cortical bone and limited (20% to 30%) reductions in the risk of non-vertebral fractures. Raloxifene has no effect on the risk of non-vertebral fractures.

The only available treatment that stimulates bone formation, parathyroid hormone, also increases bone resorption. The increased formation wanes over 12 to 18 months of use. It improves bone mineral density (BMD) perhaps more than do antiresorptive agents and reduces the risk of vertebral and non-vertebral fractures. However, its cost and need for injection have substantially limited its use.

Each medication has adverse effects. Denosumab has only been approved for 1 year in Canada and long-term data (>5 years) are lacking; it is a biologic agent and concerns have been raised about an increased frequency of serious infections [7]. A randomized, controlled trial (RCT) of the bisphosphonate alendronate reported that at 2 years 60% of participants were adherent [8]. Bisphosphonates, if taken improperly, can cause gastrointestinal irritation and esophageal ulceration [9,10]. In addition, bisphosphonates cannot be prescribed for patients with impaired renal clearance, yet these patients are at particularly high risk for fractures [11]. More recently, there have been concerns about the occurrence of fractures in the lateral femur, so-called atypical fractures, associated with the use of bisphosphonates for more than 3 years [12]; in response to this, concern Health Canada has mandated a warning about atypical fractures be placed in the product label for all bisphosphonates. Estrogen (with or without a progestin) reduces the risk of all types of fractures by 25% to 33%, but with risks that outweigh its potential benefits for fractures in the vast majority of women [13]. Raloxifene is a weak antiresorptive that does not decrease the risk of non-vertebral fractures [14]. It is associated with a three times higher rate of thromboembolic disease, an increase in hot flushes, leg cramps, leg swelling, and an influenza-like syndrome [14,15]. Parathyroid hormone is prescribed as a daily subcutaneous injection, and cannot be used for longer than 24 months in part due to concerns of osteosarcoma [16].

In addition to the adverse effects, most of these treatments are expensive: parathyroid hormone can cost upward of CA$30,000 for a 2-year course of treatment and the antiresorptive agents (with the exception of estrogen and alendronate) cost more than CA$700/year, are either unavailable or unaffordable outside of North America and Western Europe, and of uncertain safety when used long term (>10 years).

The limitations of the current therapies have fuelled interest in alternatives. An optimal agent would be one that decreases bone resorption while also increasing bone formation, BMD, cortical bone mass, and with potential to decrease non-vertebral fractures more than do existing treatments. It would be convenient to take, inexpensive, have minimal adverse effects, be safe for long-term use, and would be available worldwide.

Current treatments have similar and limited mechanisms of action. In usual bone remodeling, resorption by osteoclasts signals, and is coupled with, subsequent bone formation by osteoblasts (thus bone turnover is coupled); generally speaking, bone turnover occurs at a higher rate in trabecular bone (the spine) than in cortical bone (the hip). All the antiresorptive agents decrease bone resorption but also decrease bone formation, and because they have greater effects at sites of highest turnover, they have a greater impact on trabecular versus cortical bone. The mode of action of antiresorptives has at least two important clinical implications: there is a ceiling with regards to the amount of bone that can be gained and antiresorptives predominantly decrease only the risk of vertebral fractures, which occur in trabecular bone [17,18]. However, non-vertebral fractures which typically occur in cortical bone (including fractures of the hip, legs, upper arms and forearms) account for most of the morbidity, mortality and costs due to fractures [19] and even the most potent antiresorptive drugs reduce the risk of non-vertebral fractures by less than one-third [7,20-24]. Parathyroid hormone (PTH) is the only therapy that increases bone formation (but it also increases bone resorption). It reduces vertebral fracture risk by about 70% and may decrease the risk of some cortical fractures, but has not been shown to reduce the risk of hip fractures [16].

To decrease the disability, death and medical costs due to osteoporosis it is essential to identify an inexpensive, widely available treatment that both increases bone formation and decreases bone resorption and thereby increases cortical thickness and the bending strength of largely cortical bones. We believe that nitric oxide, in the form of organic nitrate, may have many of these attributes.

**Figure 1:**
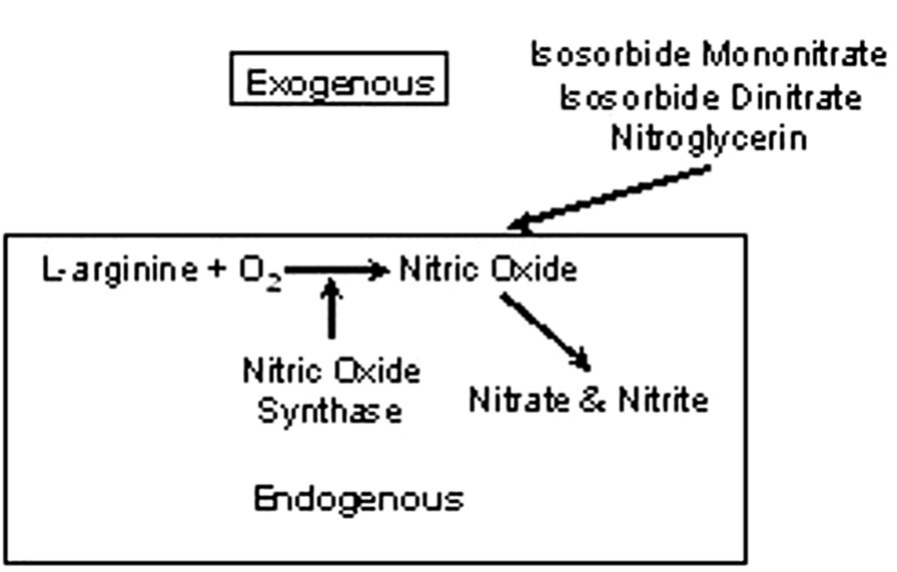
Sources of nitric oxide.

Nitric oxide (NO) is a short-lived free radical involved in the regulation of many physiological processes, including bone remodeling [25,26]. There are three sources of NO (Figure 1). NO can be generated by the nitric oxide synthase (NOS) enzymes from molecular oxygen and the terminal nitrogen of the amino acid l-arginine [25,27]. NO can be generated non-enzymatically from nitrite in the acid environment of the stomach, and organic nitrates (for example, nitroglycerin (NTG), isosorbide mononitrate (ISMO), isosorbide dinitrate) can act as NO donors [28].

### Research to date

Data from both cellular and animal studies support the use of nitric oxide for treatment of postmenopausal osteoporosis. Nitric oxide can inhibit osteoclast activity and acts as a signaling molecule in osteoblasts and osteocytes [29-35]. *In vitro* studies demonstrate that NO has a biphasic effect on osteoclast activity and bone resorption [29,32,33,35,36]; low concentrations potentiate bone resorption while high concentrations inhibit activity [30,34,37]. The effects of NO on osteoblasts are less well characterized. Some, but not all, studies report that low concentrations of NO stimulate osteoblast growth and differentiation [38]. Further, mice lacking nitric oxide synthase have defective bone formation due to defects in osteoblast differentiation and function [39,40], indicating that NO plays a key role in regulating bone formation.

NTG ointment, an NO donor, prevents bone loss in rats [41]. Ovariectomized rats were treated with vehicle, 17-β estradiol, NTG ointment, or a combination of 17-β estradiol and NTG. Compared with baseline, treatment with NTG increased BMD in ovariectomized rats (mean ± SD = 25 ± 2% to levels similar to those found in sham operated rats (mean ± SD = 25 ± 2%) and the increase in BMD in ovariectomized rats treated with NTG ointment (mean ± SD = 20 ± 3%) was greater than the increase in ovariectomized rats treated with vehicle (mean ± SD = 8 ± 3%) (Additional file 1). This suggests that nitrates, which act as NO donors, might preserve or even increase bone mass. Supportive of this concept are data from observational and randomized trials, reviewed below.

In 1998, we began investigating the relationship between the use of nitrates and BMD in humans using data from the Study of Osteoporotic Fractures (SOF), a multicenter, prospective, observational study of 9,704 ambulatory, Caucasian women, aged 65 years and older [42]. We hypothesized that women taking nitrates intermittently would have significantly higher bone mass than those who took nitrates continuously. Continuous exposure to organic nitrate causes tachyphylaxis to its vascular effects. Data from the cardiovascular literature report tachyphylaxis to nitrates with increasing frequency of dose [43,44]. Tachyphylaxis to nitrates may develop in bone: rats given NTG ointment daily for 12 weeks had increases in BMD similar to those with estrogen, yet more frequent administration abolished any beneficial effects (Additional file 1) [45].

We compared hip and heel BMD among nitrate users (391 women) and non-users (5,827 women) identified by self-report. Women who reported using ISMO, isosorbide dinitrate, or NTG more than once a day, every day, were classified as continuous users (n = 317), and all other women were classified as intermittent users (n = 74). Compared with non-users, nitrate users were more likely to have risk factors for low BMD [46]. After adjusting for these differences, and for estrogen use, we found that hip BMD was 2.6% higher and heel BMD was 5.3% higher among intermittent nitrate users compared to non-users, and intermittent nitrate users had greater BMD than continuous users at both these sites (Additional file 1). The results were consistent with our hypothesis that intermittent use of nitrates improves bone mass while continuous nitrate use may lead to tachyphylaxis.

There are two alternate explanations as to why intermittent nitrate use is associated with greater BMD than continuous use. First, women who use nitrates intermittently may have better health and fewer risk factors for low BMD than women who require continuous nitrates. However, adjusting for known differences in health status did not mitigate the nitrate effect. Second, the findings may be due to chance variation. However, the results were robust and statistically significant when we examined BMD at both the hip and the heel.

The mean dose of nitrate among women reporting intermittent use was 0.2 mg/day of NTG. This is well below the doses required for angina treatment: a typical single dose is 0.3 mg and daily doses vary from 0.3 mg to 0.9 mg. Among the 74 women reporting intermittent use, the type (ISMO, isosorbide dinitrate and NTG), the form (sublingual tablet or spray, oral tablets, sustained release tablets, transdermal patch, or ointment) and the dose varied.

Of note, there have been two previous case-control studies that have reported on the effect of organic nitrates on fracture risk [47,48]. Both of these studies demonstrate that nitrate use was associated with a decreased risk of fracture, including hip fracture (by about 10% to 15%), and that the reduction in fracture risk was greatest among those using low-dose nitrates on as needed basis. However, these studies were unable to compare formulations with regard to efficacy or headaches.

These findings, considered together with findings from our previous study [42], suggest an intriguing possibility: that nitrates of any formulation administered intermittently can increase BMD and decrease the risk of fractures, particularly the risk of non-vertebral fractures. However, we have not identified the most potent treatment and one that causes headaches least frequently. This research proposal will make these comparisons and set the stage for a trial to test the antifracture efficacy of nitrates.

The next step in our program of research was a RCT comparing the effects of placebo and intermittent ISMO on markers of bone turnover in postmenopausal women. We randomly assigned 144 women (≥3 years postmenopausal with femoral neck BMD T-scores between 0 and -2.5) to 12 weeks of placebo or intermittent ISMO of 5 mg or 20 mg per day; typically, ISMO is prescribed at 20 mg twice a day. We measured changes from baseline in urine N-telopeptide (NTx), a marker of bone resorption and serum bone-specific alkaline phosphatase (BSAP), a marker of bone formation [49].

**Figure 2:**
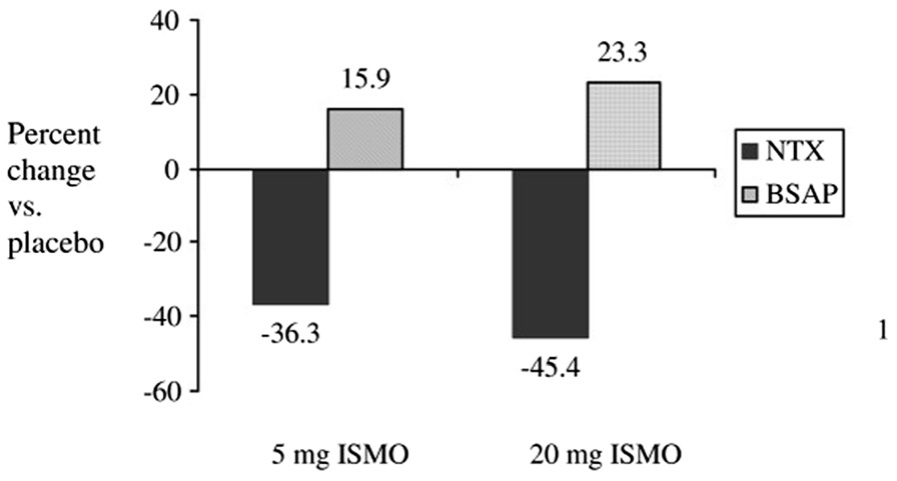
Percentage change in urine N-telopeptide (NTx) and serum bone-specific alkaline phosphatase (BSAP) in women randomly assigned to 5 or 20 mg of isosorbide mononitrate (ISMO) daily compared to women assigned to placebo.

Our earlier work suggested that the effect of nitrates on bone was a class effect and as such we did not think the formulation nitrate we chose would result in substantially different effects on markers of bone turnover as long as it was given intermittently and in a low dose [42]. We chose to study ISMO because it is completely and consistently absorbed, does not have a first-pass effect, has linear dose-dependent pharmacokinetics, and marked dose-dependent hemodynamic effects [50]. We chose doses of 5 mg and 20 mg; pharmacologic data demonstrate that the threshold of oral activity of ISMO is 5 mg and the maximum response is reached with doses of 20 mg [43]. To prevent tachyphylaxis [44], we gave ISMO, which is typically administered twice a day, once a day or intermittently. We found that, compared with placebo, women randomized to intermittent ISMO at 20 mg had a 45.4% decrease in NTx (95% confidence interval (CI) 25.8 to 64.9) and a 23.3% increase (95% CI 8.9 to 37.8) in BSAP. Women randomized to intermittent ISMO at 5 mg had a 36.3% decrease in NTx (95% CI 14.8 to 57.8) and a 15.9% increase in BSAP (95% CI 1.1 to 30.7) (Figure 2) [49].

The decreases in NTx that we observed with 20 mg of ISMO are similar to those reported with alendronate, risedronate, and estrogen (about 50%) and greater than the 25% decreases reported with raloxifene [51,52]. However, all of the antiresorptive agents concomitantly decrease rates of bone formation. In contrast, we observed that treatment with ISMO resulted in significant increases in BSAP.

The decrease in resorption, coupled with the increase in formation, suggests that ISMO may reduce fracture risk to an even greater degree than that seen with the current antiresorptive agents. The only adverse event was headache. Headaches were more common among women randomized to ISMO (5 and 20 mg groups combined n = 55, 57%) compared with placebo (n = 2, 4%; *P* = 0.004). Headaches were no more common among women taking 20 mg of ISMO (50.9%) than among women taking 5 mg of ISMO (49.09%; *P* = 0.7).

Only one other randomized trial has examined the effects of nitrates on bone. This was an open-label trial that randomized 16 oophorectomized women, aged 36 to 45 years, to intermittent NTG ointment (15 mg/day) or oral conjugated estrogen (0.625 mg) [41,53]. After 6 months, women taking NTG ointment had a 40% decrease in NTx and 25% increase in BSAP compared with baseline. The magnitude of change in bone turnover markers was similar to what we found with ISMO: consistent with the concept that the effects of nitrates on bone might be independent of the formulation. The number of headaches was not reported, however personal communication with the primary investigator (Dr. Sunil J Wimalawansa) indicted that there was no dropout due to headaches in this study.

The magnitude of effects on bone markers and the uncoupling between formation and resorption was similar for both NTG and ISMO. The lack of headaches with NTG deserved further study and this served as the basis for our next research project: ‘A CIHR funded study with two placebo controlled trials: a four week pilot study and a 27 month main study in healthy postmenopausal women’. The aim of the pilot study was to determine the best tolerated preparation of nitrate for future studies. Specifically, we assigned 22 subjects to intermittent NTG at 15 mg/day and intermittent ISMO at 20 mg/day, each for 1 week. The order of the treatments was random, accompanied by a placebo control (identical in sight and smell to the active treatment). Inbetween each treatment, there was a 2-week washout period. Subjects recorded the severity of headaches upon awakening every day for 4 weeks using a visual analogue scale (VAS). We calculated the mean headache score for each subject over both 7-day treatment periods and then the mean headache score, considering all subjects for each of the NTG and ISMO treatment periods. We found that the ISMO was associated with more frequent headaches (12 women reported daily headaches vs 5 women) and more severe headaches than the NTG ointment (mean headache score: 4/5 for ISMO vs 2/5 for NTG ointment) [54]. As a result, we used the NTG ointment for our next study reviewed below [1]. To limit differential dropout due to headaches, the main trial followed from a 1-week nitrate run-in phase; women who discontinued the nitrate due to headaches did not enter the main trial.

Our trial was designed to test the effects of nitroglycerin on bone turnover, density, geometry, and strength. We randomly assigned 243 postmenopausal women with BMD T-scores between 0 and -2.0 at the lumbar spine to 15 mg of NTG ointment or placebo daily at bedtime for 24 months. We examined four outcomes: BMD, pQCT, bone turnover and headaches. We found that at 2 years, compared with placebo, NTG increased spine BMD 6.7% (95% CI 5.2 to 8.2; *P* <0.001), femoral neck BMD 7.0% (95% CI 5.5 to 8.5%; *P* <0.001) and total hip BMD 6.2% (95% CI 5.2 to 7.3; *P* <0.001) [54]. This pattern is unique. All current treatments improve spine BMD (largely trabecular) more than femoral neck BMD (largely cortical). These results suggest that NTG has uniquely potent effects on cortical bone mass that may translate into greater effects on the risk of non-vertebral fractures.

We also found that at the radius and the tibia, respectively, 2 years of NTG increased cortical thickness (13.9% and 24.6%), cortical area (10.6% and 10.0%) and periosteal circumference (7.4% and 2.9%), with small effects on cortical density. This pattern indicates that nitroglycerin increases cortical bone mass; the increase in periosteal diameter suggests that it may induce formation of new bone on the periosteal surface, a biologically unique effect not observed with antiresorptives. As would be expected from these effects on cortical bone, NTG increases indices of strength: section modulus or bone bending (10.7% and 9.8%), and polar moment of inertia or bone twisting (7.3% and 14.5%) at the radius and tibia respectively [54]. These improvements in bone strength indicate that NTG should decrease the incidence of non-vertebral fractures.

NTG ointment uncoupled bone formation from bone resorption. At 2 years, the BALP levels increased by 36% and the NTX levels decreased by 51%, the results being statistically significant [1]. Unlike treatment with PTH, the magnitude of bone formation continued to increase over 2 years.

The most common adverse effect we observed was headache. During a 1-week run-in phase with 15 mg of NTG ointment, 104 of 400 women stopped treatment because of headache. Among those who continued into the randomized trial, seven in the NTG and two in the placebo group stopped treatment during the 2 years because of headaches. Although 36% of women receiving NTG reported a headache during the first 6 months of the trial, headache was uncommon by 2 years (1.7% in the nitroglycerin vs none in the placebo group).

Of note, a recent randomized trial of once-daily NTG ointment (Nitro-Bid 22.5 mg) did not find increased BMD at the lumbar spine, femoral neck of total hip. However, adherence to treatment was poor and there were additional concerns about the quality control of the bone density endpoints [55].

Considered together, our data, even in the light of the negative study suggest that changes in cortical bone geometry caused by NTG ointment may translate into an important reduction in fractures: non-vertebral fractures, such as hip fractures, in particular. Further, there might be an even greater risk reduction in all fractures compared with the currently available osteoporosis treatments. However, before proceeding with a large randomized controlled trial to determine if nitrates can reduce fractures we need to conduct a dose ranging study that will lead to the identification of a nitrate preparation that is easy to use and gives the lowest frequency of headaches together with the greatest changes in bone turnover markers. This is the purpose of the current research proposal.

## Methods and design

### The proposed trial

This will be an open-label randomized trial comparing five formulations of nitrates for their effects on bone turnover markers and headache. Because we will compare our results to our previous CIHR funded trial of NTG ointment, we have designed the trial to be as similar as possible to that earlier trial in study location, enrolment of the subjects, and choice of endpoints. We will recruit postmenopausal women age 50 years or older with no contraindications to using nitroglycerin (NTG). Our trial will consist of two phases. A run-in phase and a treatment phase, outlined below.

There are at least 13 nitrate formulations, each with varying doses. The comparative effects of available nitrate formulations have not been studied. Their effects on bone formation and resorption have not been compared. Their effects on headaches, the most common adverse effect, have not been evaluated and there are no data comparing the pharmacokinetics and little laboratory work relevant to selection of preparation or dose. Cellular, animal and human data suggest that effect of nitrates on bone is independent of formulation, but the dose should be low and the formulation used intermittently. However, it is possible that there might be some formulations that are preferred over others and the number of headache may vary by formulation. As such, before proceeding with a fracture trial, it is critical to identify the nitrate formulation that gives the greatest effects on bone turnover, with the fewest headaches, and is easy to use.

In selecting the formulations and doses to be tested in the current study we consulted with a group of scientists, including a cardiologist, a scientist who conducts cellular research in the area of nitric oxide, an internist, and a pharmacist/chemist. Our selections of formulations and dose were guided by several considerations. First, although we found strong effects of NTG ointment on bone, it is not an ideal preparation: approximately 2.5 cm (1 inch) is squeezed onto paper that is then taped to the skin. Participants complained of messiness, the paper sometimes falling off during sleep, and the dose being varied with different amounts squeezed onto the paper. Second, we include the 20 mg tablet of ISMO because it is convenient: we have previously shown that it increases markers of formation and decreases makers of resorption, and while it was a different study with a different design, the dropout due to headaches among women randomized to ISMO (20%); far less than what we observed during the run in phase with NTG ointment (about 40%) [48,54]. Third, we will use formulations that last 8 h or less because the sensitivity to effects of nitrates decreases with increasing frequency of dosing and continuous use (tachyphylaxis) [44]. Our data from the SOF found that women using nitrates intermittently had had significantly higher BMD than those who took them continuously [41]. Fourth, we have included sublingual tablets that produce a rapid peak effect because of the possibility that agents that induce bone formation might have greater effects if they rapidly reach peak levels with rapid offset, as has been shown for teriparatide [15]. This formulation was the most common one used by women taking intermittent nitrates in the SOF study. Fifth, we included a patch because it may have similar absorption of NTG as ointment but avoids the messiness. To preserve the 8-h duration, and minimize tachyphylaxis, we chose the highest-dose patch but will ask subjects to remove it by 8 versus 12 h. Finally, we will give the nitrate at bedtime as our earlier work demonstrated that taking the nitrate at bedtime resulted in a lower frequency of headaches than when taken in the morning.

We will start with a run-in phase to exclude women who would be unable to start the main trial because of headache. We will enroll about 420 women in the run-in phase, each to receive all of the 5 potential treatments (see below) in random order for 2 days each with 2-day washout period between treatments for a total of 18 days. The washout exceeds 10 half-lives of all of our formulations (which vary from 32 minutes in the case of sublingual NTG to 8 h for transdermal ointment) and will eliminate any carry-over effects [56-59].

Subjects will be seen at the start of the run in phase. We will take blood and urine for markers of bone turnover. Subjects will be given written and verbal instructions concerning the order of their treatment assignments, duration of the wash-out period and how to complete the VAS. We will also call subjects once at the start of the run in phase to confirm that the instructions have been understood and are being followed.

Those who tolerate all five formulations will enter the treatment phase and be randomly assigned to one of six groups: 0.3 mg sublingual nitroglycerin tablet, 0.6 mg of the sublingual tablet, a 20 mg tablet of isosorbide mononitrate, a 160 mg nitroglycerin transdermal patch (to be used for 8 instead of 12 h), and 15 mg of the NTG ointment used in the previous trial. To assure that the NTG ointment has the effects observed in our previous trial, we will also include a sixth randomized group to receive matching placebo ointment applied at night for about 8 h. All women will be randomized to receive one of the five formulations (or placebo) according to the randomization list provided by the study pharmacist (Sunnybrook Hospital, Toronto, Ontario, Canada). Women receiving the ointment formulation are blinded on whether they received the active NTG ointment or placebo. They will be unblinded, if they wish, upon study completion. We will continue enrolment until we have randomized 210 women, about 35 women per group. Concentrations of bone formation (BALP and P1NP) and bone resorption (CTX and NTX) will be measured in samples taken at study entry (at the start of the run in phase) and at 12 weeks. Subjects will record the number and severity of headaches on a daily basis during the run-in phase and then weekly after that. According to the product monograph, other possible events that are reported less frequently include: postural hypotension (lightheadedness going from lying/sitting to standing); increased heart rate; faintness; flushing; dizziness; nausea; vomiting and dermatitis (skin redness or irritation). To date, in our clinical trials we have not noted these less frequent adverse events. However, we will monitor for these effects.

Subjects will be seen again at the start of the treatment phase. At this visit, and as we did in our previous trial, we will administer the Block food-frequency questionnaire [60] to estimate dietary calcium and vitamin D intake and if required provide supplemental calcium carbonate (in 500 mg tablets) and vitamin D in 400 IU tablets as needed so that the total daily calcium intake for all study subjects will be 1,200 mg and the vitamin D intake will be 800 IU; these are the intakes recommended in the recently published guidelines for the prevention and treatment of OP in Canada [61,62]. Calcium and vitamin D are the mainstay of all treatment regimens for OP and any new agent should be evaluated to assess the additional benefit it would provide. Further, by providing calcium and vitamin D, we hope to discourage subjects who, after being enrolled in an ‘OP trial’, start taking additional, unreported calcium and vitamin D (cointervention). Calcium and vitamin D will be prepared and packaged by the study pharmacist (Sunnybrook Hospital, Toronto, Ontario, Canada), and instructions on how to take the supplements and the PI’s office number will be printed on each bottle. Subjects will be given standard verbal instructions by the research assistant and reminded to take the calcium and vitamin D daily with the morning meal. We will also provide the subjects with their treatment assignment and provide standard written (on the package and a information sheet) and verbal instructions on how to take the treatment. The treatment assignment will be prepared and packaged by our study pharmacist and the PI’s office number will be printed on each package.

Subjects will be called twice during the treatment phase: at 4 and 8 weeks after their visit. During these calls we will ask about the use of any new prescription and non-prescription medications; we will specifically ask about the use of any drugs for the treatment of OP, and about the use of calcium and vitamin D supplements. We will assess for possible adverse effects using a standardized, interviewer-administered questionnaire [63] (Additional file 1). We will first ask about any general adverse events. We then will ask specifically about headache, nausea, and dizziness (the three most common side effects associated with nitrate use [42]), and subjects who drop out due to nitrate-related adverse events will be followed daily until the adverse events have resolved. We will also ask about emergency room visits, hospitalizations, and visits to walk-in clinics (defined as severe adverse events), and the reason for these visits. The questionnaire we plan to use for assessment of adverse events was developed and validated in healthy postmenopausal women as part of SAJ’s doctoral thesis [64]. Serious adverse events will be reviewed by the DSMB and decisions about continuing with the treatment assignment will be made on a case-by-case basis by the DSMB. We will see subjects at the end of the treatment phase and at this visit we will collect and count unused medication, calcium and vitamin D and administer our adverse event questionnaire. Our study design is illustrated in Additional file 1.

Our study pharmacist will use a computer-generated random number sequence to match each study identification number to treatment assignment for both the run in phase and the treatment phase.

Ideally, to protect against sources of bias, all of the treatments should be blinded. However, placebos for all formulations are unavailable, and it is not feasible to prepare new ones for this study. The primary outcome of change in markers of bone turnover is unlikely to be influenced by unblinded use of the formulations; note that technician who measures our markers will be blinded to treatment assignment. We recognize that reports of headaches are subjective and might be influenced by knowing the treatment assignment: a woman taking an ‘active’ drug might be more likely to notice headaches. We will note this potential bias as we consider the selection of the best preparation.

The run-in phase and treatment phase will have identical inclusion and exclusion criteria (Additional file 1). As in our previous trial, we will include women aged 50 years and older whose last menstrual period occurred at least 3 years ago. Those without a uterus will be eligible after age 55 years.

We will use the same exclusion criteria as in our previous trial: (1) women with a history of previous fracture; (2) those who report a diagnosis of osteoporosis (‘osteopenia’ will not be excluded) (note that we did consider measuring BMD at entry to the study to exclude women with low BMD as it is not feasible within the budget; if a woman has low BMD not identified at study entry, her risk of fracture during a 12 week trial would be very low); (3) a history of disorders such as hyperparathyroidism or Paget’s disease; (4) treatment within 12 months of study entry with any agent that may influence bone metabolism including any hormone, antiestrogen or raloxifene, prednisone (equivalent to 5 mg/day for 12 months or greater), lithium, or serotonin reuptake inhibitors; (5) treatment with any oral bisphosphonate, including alendronate, risedronate or etidronate use for at least 4 weeks, within the last 3 years and any previous treatment with the very long-lasting intravenous zoledronate; (6) treatment with parathyroid hormone or denosumab within the past 12 months; (7) current treatment with nitrates; (8) any history of migraine headaches (nitrates can exacerbate migraines); (9) history of angina or cardiovascular disease; (10) inability to give informed consent; and (11) hypersensitivity to nitroglycerin.

### Outcomes to be assessed

#### Run-in phase

During the run-in phase, subjects will receive, in random order, each of the five nitrate formulations for 2 days with a 2-day wash out period between formulations. We anticipate potential dropouts of participants both during the run-in and prior to the intervention phase, with main reason for discontinuing the study being headaches. We will immediately discontinue participants who have severe headaches with any of the formulations as a contraindication for any use of nitrates. To maximize efficiency and minimize dropouts, participants will be able to continue into the treatment phase immediately upon completion of the run-in phase.

Subjects will record the severity of headaches upon awakening every day during the run-in phase using a VAS (Additional file 1). The VAS has documented reliability, can detect subtle changes in headache and has been used in several studies, including our earlier CIHR funded study, designed to study the effects of NTG ointment on headache in healthy women [54,58,59]. To help establish the preferred preparation, every participant will be asked to rate and rank the five formulations and write comments about their acceptability for long-term use. We will also use these data together with the mean headache score (for each subject over the treatment periods and then the mean headache score considering all subjects in each of the treatment groups), to determine which formulations, if any, will not be continued in the treatment phase. As we aim to assess treatment options with reasonably broad acceptability, if there is one formulation that gives headaches in more than 75% of subjects in the run-in phase in the first 6 months of recruiting or is uniformly disliked in terms of mode of application, then we will eliminate that arm from our intervention phase. However, previous studies suggest that rate of headache will not be this high and individual preferences are likely to vary enough to merit consideration of effect, and we anticipate carrying forward all formulations to the intervention phase.

### Treatment phase

We will determine the percentage change, from baseline, in two markers of bone resorption (CTX and NTX) and two markers of bone formation (BALP and P1NP) among each of the five treatment arms and the placebo arm; we measured BALP and NTX in our previous study and the International Osteoporosis Foundation has now proposed that all trials include P1NP and CTX [65]. All four markers will be measured on samples drawn at the start of the run-in phase, as it is possible that there will be an effect on bone turnover with the short bursts of nitrate treatment during the run-in phase, and again at the final 12-week visit. We will measure CTX by electrochemiluminescence immunoassay (Roche Elecsys, Roche Diagnostics, Germany); the interassay coefficient of variation (CV) is 4.7%. We will measure NTX by enhanced chemiluminescence immunoassay (Vitros ECi, OrthoDiagnostics, USA); the interassay CV is 7.9%. P1NP will be measured by electrochemiluminescence immunoassay (Roche Elecsys, Roche Diagnostics); the interassay CV is 9.1%. BALP will be measured by paramagnetic particle immunoassay (Access 2, Beckman Coulter); interassay CV: 7.5% [66]. To minimize variability, we will collect fasting serum samples and second morning urine samples, store them at -70°C, and analyze all the samples together in a single laboratory at study completion.

Note that bone turnover markers allow us to assess the effects of treatment by 12 weeks. We recognize that bone markers are biomarkers for the clinical outcome of interest (fracture). However, a phase II trial of dose selection using change in BMD or bone geometry as endpoints would be prohibitively large, long, and expensive. Further, recent data suggest that higher levels of some bone resorption markers predict fractures independently of BMD [67,68] and with antiresorptive therapy greater treatment-related decreases in bone turnover are associated with a decreased risk of vertebral and non-vertebral fracture [69,70].

Note that all our trial protocol has and will continue to be conducted in accordance with the Helsinki declaration [71]. Our protocol has been reviewed and approved by the appropriate institutional research ethics boards (Women’s College Hospital and The University of Toronto) and all subjects are required to provide written informed consent before participating in our study.

### Sample size and analyses

We will use the ‘multiple comparisons with the best’ (MCB) approach for data analyses and determination of sample size [72]. We will give primacy to differences in bone formation (measured as percentage change over baseline) because this is the unique effect of nitroglycerin and the one most likely to reduce the risk of non-vertebral fracture. We wish to be able to estimate differences between formulations in percentage change over baseline to within plus or minus 15% assuming a standard deviation of percentage change of 25%. These assumptions are based on prior studies of bone turnover markers that have reported a minimal of 15% change in response to treatment and similar changes in SD [64,73-77].

At the analysis stage, MCB guarantees that differences between preparations will be estimated to within plus or 15% with 95% confidence (over all the comparisons made). These intervals result in one of the following conclusions: (a) a preparation is not the best (that is, does not increase formation the most), (b) a preparation is better than all the rest, or (c) the preparation might be best and is within 15% of the best.

At the design stage, MCB guarantees that if the best preparation is at least 15% better than the next best preparation, it will be ranked as best with probability at least 0.95. Based on these considerations, we require 30 subjects per group or 180 subjects total complete the study. We assume a dropout of rate of 10% post randomization, so we will enroll 210 subjects, approximately 35 per group. If one preparation is determined best by MCB, we will select that preparation. However, a possible result is that multiple preparations will be within 15% of the best. In that case we will rank preferred formulations based on their acceptability and tolerability; that is, the ISMO tablet may be preferred for its convenience and familiarity if it is nearly as good as the best. Thus, MCB allows consideration of other factors in deciding the preparation to be selected. Our MCB strategy allows practical consideration of ease-of-use and tolerability to guide selection of the preparation for future studies. If two or three preparations have similar effects on bone formation (do not differ by at least 15%), we will use tolerability data and subject preference from the run-in and previous studies to guide our choice. We expect the decision may benefit from the input of experts in osteoporosis and note that additional studies might be useful to select an ideal preparation and dose.

Our sample size was determined using STATA V. 10 on the basis of assessing the treatment effect. However, there is some likelihood we will need to consider other variables if there are multiple treatments that are within 15% of the best. The limiting factor in comparisons will be headache prevalence, but with given sample size (420 in the run-in) we will have power of at least 0.8 to detect a difference in prevalence of 10% with α = 0.05.

### Subject recruitment and compliance

We will recruit 420 subjects for our run-in phase. Based on our prior experience (see below) we anticipate that 50% of these will continue on to the treatment phase. We anticipate recruiting the 420 subjects for the study over 24 months (about 4 subjects per week). As we did for our previous, successful trials, we will advertise in local newspapers, on local radio and television stations, and place flyers in doctor’s offices and OP clinics. Potential subjects will be assessed for eligibility by telephone. Subjects who meet eligibility criteria and are interested in participating after the telephone interview will come to the study center where they will receive written information about the study. We have established recruitment goals and timelines that are feasible based on prior experience. Our previously funded CIHR RCT recruited four subjects per week. We have maintained these goals for the current proposal. We are confident that we can meet this goal for several reasons. Based on our previous study we have identified methods of highly successful and efficient recruitment; for example, mailing study information letters out to women who have had bone mineral density tests at Women’s College Hospital, a leading site for osteoporosis research at the University of Toronto. Furthermore, our previous study has generated national and international interest in the lay press (television interviews on CTV and radio interviews on CBC’s Metro Morning) and we will use our contacts with the press to assist with publicizing our new study. In addition, all our collaborators have osteoporosis clinics and will assist in subject recruitment. Finally, and perhaps most importantly, we will engage both a research assistant and research associate; hiring an adequate number of personnel is critical to ensure timely and efficient recruitment and enrolment of study subjects.

As with our previous studies, we will collect and count medication to assess compliance. Pill counts are a simple, inexpensive method of monitoring medication compliance and are well correlated (r = 0.69) with electronic monitoring devices [78]. In our trials, women who had more than 15% of any pills remaining were classified as non-compliant.

In our RCT of isosorbide mononitrate, most of the 144 women were compliant with calcium and vitamin D in the pretreatment phase; 25 women had more than 15% of calcium pills remaining and 26 women had more than 15% of vitamin D pills remaining. Non-compliance in the treatment phase was higher, 12 of 48 women (25%) were non-compliant with placebo and 29 of 96 women (34%) were non-compliant with the intermittent nitrate. Women who were non-compliant were four times more likely to report headaches than women who were compliant [63]. There were 25 dropouts due to headaches (20 of the 96 randomized to ISMO (21%) and 5 of 48 randomized to placebo (10%) and 6 of the 144 (4%) women did not return to the study center after randomization and were lost to follow-up [48].

Our RCT of NTG ointment recruited 400 women to the run-in phase and 243 entered the main trial; this design (a run-in phase followed by a treatment phase) is similar to our current study. Note that 65 of 157 (41%) of subjects did not enter the main trial due to headaches. Of the 243 women who were randomized 10 of 126 (8%) participants assigned to NTG and 8 of 117 (7%) subjects in the discontinued treatment and 2 subjects in each of the NTG (2%) and placebo group (2%) were lost to follow-up [54].

Based on our previous experience (data presented above) and the experience of our coinvestigators, we estimate a 40% dropout rate due to headaches during the nitrate run-in phase, and a 10% loss to follow-up/non-compliance rate; this latter estimate is conservative given that the study is only 12 weeks in duration. Thus, we will ‘over-recruit’ by 100%; specifically, we will recruit 420 subjects of whom we anticipate that 210 subjects will complete the run-in phase and enter the treatment phase (35 per group).

This will be a multicenter trial that will recruit from five sites: The University Health Network, St Michael’s Hospital, Women’s College Ambulatory Care Centre, Sunnybrook Health Sciences Centre, and the Hamilton Health Sciences Centre at McMaster University. Our statistician coinvestigator is based at McGill University, and SRC is based in San Francisco. As well, we will confer with national and international experts on the conduct of the trial and the interpretation of our data.

### Trial management

SAJ (the primary investigator) will oversee the day-to-day management of the trials. Two individuals (a research assistant and a research associate) will be responsible for the day-to-day management of the study from ethics submissions; subject recruitment, consenting and enrolment; to spinning, aliquotting and storing blood and urine; coordinating medication/placebo distribution with the research pharmacist; data entry and cleaning of data; and managing the study’s expenses (financial account). This arrangement is similar to our previous, successful RCTs of nitrates [48,63].

We will also create a DSMB. All adverse events and clinically important medical conditions will be recorded and faxed to the committee for adjudication. Subjects will be discontinued from the study if they develop clinical fractures or if they develop medical conditions that necessitate starting nitrates (for example, developing angina).

## Discussion and conclusions

The number of osteoporotic fractures is increasing worldwide as the population is aging. Current treatments for osteoporosis are limited by cost, side effects and most importantly efficacy. Current treatments decrease vertebral fractures (which consist of trabecular bone) but have very limited effects on cortical bone, yet most osteoporotic fractures occur at these sites (for example, the hip, legs, forearm and upper arm). There is a need for easily administered, inexpensive, well-tolerated agents that increase bone cortical strength and substantially decrease the risk of fractures. Our data suggest that organic nitrates may meet this need.

A randomized trial with fracture endpoints is essential to establish the efficacy of nitrates for clinical use. To design and conduct a fracture-prevention trial requires choosing a preparation and dose of nitrate that maximizes its beneficial effects on bone while minimizing adverse effects (headaches). In the current study, we will compare five widely available nitrate formulations for the efficacy on markers of bone formation and resorption and for the number and severity of headache they cause. We will use this information together with the input of experts in the treatment of osteoporosis to select a formulation and dose of nitrate to study in a fracture-prevention trial. Ultimately, our research aims to decrease the burden of illness due to osteoporotic fractures.

## Trial status

This trial was funded by the Canadian Institutes of Health Research, Physicians' Services Incorporated Foundation (PSI) and California Pacific Medical Center Research Institute (CPMC RI). We are currently recruiting study subjects.

## Abbreviations

BALP: Bone specific alkaline phosphatase; BMD: Bone mineral density; IHR: Canadian nstitutes of health research; CTX: C-telopeptide; CV: Coefficient of variation; DSMB: Data Safety Monitoring Board; ERT: Estrogen replacement therapy; ISMO: Isosorbide mononitrate; MCB: Multiple comparisons with the best; NABT: Nitrates and bone turnover; NOS: Nitric oxide synthase; NTG: Nitroglycerin; NTX: N-telopeptide; OP: Osteoporosis; P1NP: Procollagen type I N-terminal propeptide; pQCT: Peripheral quantitated computed tomography; PTH: Parathyroid hormone; RANKL: Receptor activator of nuclear factor-κB ligand; RCT: Randomized controlled trial; SOF: Study of Osteoporotic Fractures; VAS: Visual analogue scale.

## Competing interests

The authors declare that they have no competing interests.

## Authors’ contributions

SAJ and SRC are responsible for the conception and design of the protocol and will be responsible for analysis and interpretation of the data. RCB and LSR will be responsible for day-to-day management of the clinical and for data acquisition. SAJ and CJH were responsible for drafting the protocol and SRC reviewed and revised it for important intellectual content. All authors have given final approval of the version to be published. Funded by the Canadian Institutes of Health Research; CIHR MOP119423.

## Supplementary Material

Additional file 1 **Appendix 1.** effect of conjugated estrogen and transdermal nitroglycerin on BMD in ovariectomized rats. **Appendix 2.** percentage change in lumbar spine BMD in ovariectomized rats after 12 weeks of treatment with transdermal nitroglycerin. **Appendix 3.** differences (mean ± SD) in BMD at the total hip and heel in nitrate users and non-users (unadjusted and adjusted for estrogen use and baseline differences). **Appendix 4.** study design. **Appendix 5.** NABT inclusion and exclusion criteria. **Appendix 6.** NABT Adverse Events Questionnaire. **Appendix 7.** NABT Visual Analogue Scale. Click here for file
