# Supplementary material for: Nitrates and bone turnover (NABT) - trial to select the best nitrate preparation: study protocol for a randomized controlled trial
Source: Trials. 2013 Sep 8;14:284. doi: 10.1186/1745-6215-14-284 (PMC3847792; doi:10.1186/1745-6215-14-284)
Supplement: Additional file 1 — Appendix 1. effect of conjugated estrogen and transdermal nitroglycerin on BMD in ovariectomized rats. Appendix 2. percentage change in lumbar spine BMD in ovariectomized rats after 12 weeks of treatment with transdermal nitroglycerin. Appendix 3. differences (mean ± SD) in BMD at the total hip and heel in nitrate users and non-users (unadjusted and adjusted for estrogen use and baseline differences). Appendix 4. study design. Appendix 5. NABT inclusion and exclusion criteria. Appendix 6. NABT Adverse Events Questionnaire. Appendix 7. NABT Visual Analogue Scale. [file 1745-6215-14-284-S1.doc]

**Additional file**

**Appendix 1.** Effect of conjugated estrogen and transdermal nitroglycerin on BMD in ovariectomized rats.

**Appendix 2.** Percent change in lumbar spine BMD in ovariectomized rats after 12 weeks of treatment with transdermal nitroglycerin.

**Appendix 3.** Differences (mean ± SD) in BMD at the total hip and heel in nitrate users and nonusers (unadjusted and adjusted for estrogen use and baseline differences).

**Appendix 4.** Study design.

**Appendix 5.** NABT inclusion and exclusion criteria.

**Appendix 6.** NABT Adverse Events Questionnaire

**Appendix 7.** NABT Visual Analogue Scale

**Appendix 1.** Effect of conjugated estrogen and transdermal nitroglycerin on BMD in ovariectomized rats [40]

| Treatment group, n = 5 for all groups | Percent increase in BMD (L2-L4) over 6 weeks |
| --- | --- |
| Sham operated | 25% ± 2% |
| Ovariectomized rats | 8% ± 3% |
| Ovariectomized + estrogen | 27% ± 5%* |
| Ovariectomized + transdermal nitroglycerin | 20% ± 3%† |
| Ovariectomized + estrogen + nitroglycerin | 22% ± 2%* |

*different than ovariectomized rats at p < 0.005

† different than ovariectomized rats at p < 0.02

**Appendix 2.** Percent change in lumbar spine BMD in ovariectomized rats after 12 weeks of treatment with transdermal nitroglycerin [44]

| Treatment Group (n = 5 for all groups) | Percent Change in  Lumbar Spine BMD |
| --- | --- |
| Sham operated | 6.3 % ± 5.3 * |
| Ovariectomy (OVX) | – 2.5 % ± 2.0 |
| OVX + estrogen | 5.9 % ± 3.4* |
| OVX + 0.2 mg transdermal nitroglycerin once a day | 6.2 % ± 2.8* |
| OVX + 0.2 mg transdermal nitroglycerin twice a day | 1.9 % ± 2.1 |
| OVX + 0.2 mg transdermal nitroglycerin three times a day | – 0.2 % ± 3.3 |

*different than ovariectomized rats at p < 0.01

**Appendix 3.** Differences (mean ± SD) in BMD at the total hip and heel in nitrate users and nonusers (unadjusted and adjusted for estrogen use and baseline differences) [41].

|  | Percent difference in BMD (95% CI) | | | |
| --- | --- | --- | --- | --- |
|  | Daily – nonusers | | Intermittent – nonusers | |
| Hip BMD |  |  |  |  |
| Unadjusted | 0 | (– 2.7 to 1.4) | 0 | (– 4.1 to 4.1) |
| Adjusted* | 1.3 | (0.14 to 4.1) | 2.6 | (0.4 to 6.8) |
| Heel BMD |  |  |  |  |
| Unadjusted | –2.6 | (– 5.3 to 0) | 0 | (– 5.3 to 7.9) |
| Adjusted* | 0 | (– 2.6 to 2.6) | 5.3 | (2.6 to 11) |

*adjusted for estrogen use and baseline differences, which are current alcohol intake, walks for exercise, physical activity, self-reported health status, falls in the past 12 months, thiazide and nonthiazide diuretic use, inability to rise from a chair independently, and frail on physical examination.

**Appendix 4.** Study Design.

**Run-in phase**: Each subject receives trial of 5 active treatments in random sequential order to assess tolerance

**Exclusion**: Patients who do not tolerate any one treatment are subsequently excluded

NTG 15 mg topical

Placebo

NTG 0.6 mg/h patch

NTG 0.6 mg SL

NTG 0.3 mg SL

**Randomization**: Subjects randomized to single treatment regimen (N=210)

ISMO 20 mg PO

Samples for bone turnover markers taken at start of run in phase

Samples for bone turnover markers after 12 weeks of treatment

**Appendix 5.** NABT inclusion and exclusion criteria.

| **Inclusion Criteria:** | Postmenopausal women aged 50 years or older. |
| --- | --- |
|  | Last menstrual period must have occurred at least 3 years prior to study entry. Women without a uterus will be eligible after age 55. |
|  |  |
| **Exclusion Criteria:** | A previous fracture of the hip, wrist, spine or ankle. |
|  | A diagnosis of osteoporosis (“osteopenia” will not be excluded). |
|  | A history of bone disorders such as hyperparathyroidism or Paget’s disease. |
|  | Treatment within 12 months of study entry with any agent that may influence bone metabolism including; hormones, anti-estrogens or raloxifene, prednisone (equivalent to 5mg/day for 12 months or greater). |
|  | Treatment with any antiresorptive agent including; alendronate, risedronate, etidronate, denosumab, used for at least four weeks within the last three years. |
|  | Previous treatment with intravenous zoledronate or parathyroid hormone. |
|  | Current treatment with nitrates. |
|  | A history of migraine headaches (nitrates can exacerbate migraines). |
|  | A history of angina or cardiovascular disease. |
|  | Inability to give informed consent. |
|  | Hypersensitivity to nitrates. |
|  | Allergies to the adhesive used in nitroglycerin patches. |
|  | Acute circulatory failure associated with marked hypotension (shock and states of collapse. |
|  | Postural hypotension. |
|  | Increased intracranial pressure. |
|  | Increased intraocular pressure. |
|  | Severe anemia. |

**Appendix 6.** NABT Adverse Events Questionnaire.

1. Ask: “Since we last spoke, have you experienced any symptoms that you don’t always feel?”

Then ask symptoms listed below:

| **Event / Symptom** | **Onset Date (dd/mm/yy)** | **Duration of Episode** | **Frequency**  **(per week)** | **Severity** | **Causality** | **Outcome** | **Ever Before Study** |
| --- | --- | --- | --- | --- | --- | --- | --- |
| Headache |  |  |  |  |  |  |  |
| Dizziness |  |  |  |  |  |  |  |
| Nausea |  |  |  |  |  |  |  |
| Vomiting |  |  |  |  |  |  |  |
|  |  |  |  |  |  |  |  |
|  |  |  |  |  |  |  |  |

**Duration**

1 = minutes 2 = hours 3 = days

**Severity**

1 = mild: does not hinder the patient’s functioning

2 = moderate: some degree of impairment to functioning, uncomfortable or embarrassing, or started medications

3 = severe: definite hazard to well-being, significant impairment of functioning or incapacitation. For example: sought medical attention, missed one or more days from work.

| **Causality re. Study Drug** | **Outcome** | **Ever Before Study** |
| --- | --- | --- |
| 1 = almost certainly | 1 = resolved | 0 = never |
| 2 = probably | 2 = improved | 1 = rarely |
| 3 = possibly | 3 = unchanged | 2 = frequently |
| 4 = unlikely | 4 = worse | 3 = continuously |
| 5 = unrelated |  |  |

Comments:

Headache_______________________________________________________________________________________________________________________________________________________________________

Dizziness____________________________________________________________________________________________________________________________________________________________________

Nausea_________________________________________________________________________________________________________________________________________________________________________

Vomiting______________________________________________________________________________________________________________________________________________________________________________

2. Ask: since we last spoke have you had any falls ?

 no  yes, **record below**

**If yes**, how many falls? ______________________________

3. Ask: since we last spoke have you had any fractures or broken bones?

 no  yes, **record below**

**If yes**, location of the fracture ______________________

4. Ask: since we last spoke have you had any emergency room visits, hospitalizations, and visits to walk in clinics ?

 no  yes, **record below**

| Date | Reason for visit | Medical record obtained |
| --- | --- | --- |
|  |  |  |
|  |  |  |
|  |  |  |
|  |  |  |
|  |  |  |
|  |  |  |
|  |  |  |

5. Ask: since we last spoke are you taking any new prescription or over-the-counter medications?

 no  yes, **record below**

Record the use of any prescription and over-the-counter medications currently being used

| **Medication Name (generic or trade)** | **Dose (mg/day)** | **PRN (yes, no)** | **Duration of use (years)** |
| --- | --- | --- | --- |
| 1. |  |  |  |
| 2. |  |  |  |
| 3. |  |  |  |
| 4. |  |  |  |
| 5. |  |  |  |
| 6. |  |  |  |
| 7. |  |  |  |

**Appendix 7.** NABT Visual Analogue Scale.

**NABT Visual Analog Scale**

Please make a vertical mark along the continuum (a straight line up and down) to indicate the severity of your headache each morning upon awakening. 0 means that you do not have a headache and 10 means that you have a terrible headache. The line is 10 cm long and the score is recorded in cm from 0 to 10.

**TREATMENT 1: ____________________**

Day 1:

0 10

(no headache) (terrible headache)

Day 2:

0 10

(no headache) (terrible headache)

*****2-day washout period*****

**TREATMENT 2: ____________________**

Day 1:

0 10

(no headache) (terrible headache)

Day 2:

0 10

(no headache) (terrible headache)

*****2-day washout period*****

**TREATMENT 3: ____________________**

Day 1:

0 10

(no headache) (terrible headache)

Day 2:

0 10

(no headache) (terrible headache)

*****2-day washout period*****

**TREATMENT 4: ____________________**

Day 1:

0 10

(no headache) (terrible headache)

Day 2:

0 10

(no headache) (terrible headache)

*****2-day washout period*****

**TREATMENT 5: ____________________**

Day 1:

0 10

(no headache) (terrible headache)

Day 2:

0 10

(no headache) (terrible headache)

*****end of run-in phase*****
